# Supplementary material for: WordCommentsAnalyzer: A windows software tool for qualitative research
Source: F1000Res. 2018 Sep 4;7:536. Originally published 2018 May 3. [Version 2] doi: 10.12688/f1000research.14819.2 (PMC6137411; doi:10.12688/f1000research.14819.2)
Supplement: Supplementary file 3 [file f1000research-7-17614-s0002.tgz › b2a79496-4ada-47c4-9682-a99ce867836d.zip!/Eight Iranian Health Journals/Archives of Rehabilitation_0.docx]

© Archives of Rehabilitation (RJ). University of Social Welfare and Rehabilitation Sciences, Koodakyar Alley, Daneshjoo Blv., Evin, Tehran, Iran. This work is licensed under [CC BY-NC 4.0](https://creativecommons.org/licenses/by-nc/4.0/).

Volume & Issue: دوره 19 - شماره 2

Title: بررسی ساختار سازه‌ای واکه‌های زبان فارسی در بزرگ‌سالان دوزبانه آذری فارسی

http://rehabilitationj.uswr.ac.ir/article-1-2271-fa.html

هدف: واکه‌ها هسته و مرکز هجا هستند. یکی از مهم‌ترین ویژگی‌های آکوستیکی آن‌ها ساختار سازه‌ای است که به جنبه‌های تولیدی و ادراکی آن‌ها کمک می‌کند. سازه‌ها نشان‌دهنده شکل و اندازه مجرای صوتی هستند، اما از آنجایی‌ که مجرای صوتی هر فردی با دیگری تفاوت جزئی دارد، سازه‌های یک واکه در افراد مختلف تا حدودی با هم متفاوت هستند. در اواخر قرن بیستم با پیشرفت علم آکوستیک و تولید ابزارهای دیجیتال، آنالیز ویژگی‌های آکوستیکی به جنبه‌های تحلیل صوت افزوده شد. تحلیل ویژگی‌ها و سازه‌های آکوستیکی منجر به ارزیابی بهتر صوت می‌شود. از آنجایی که درمان نتیجه‌بخش اختلالات گفتاری، به توانایی آسیب‌شناس گفتار و زبان در ارزیابی دقیق بیمار بستگی دارد و با توجه به اینکه سه سازه اول، از اجزای اصلی تشخیص واکه توسط شنونده به شمار می‌آیند، لذا هدف از این پژوهش، تعیین و مقایسه میانگین فرکانس سازه‌های اول، دوم و سوم واکه‌های زبان فارسی در افراد دوزبانه آذری فارسی است. روش بررسی: مطالعه حاضر از نوع توصیفی تحلیلی به روش مقطعی است. این پژوهش روی 50 نفر از دانشجویان دوزبانه (آذری فارسی) دانشکده توانبخشی دانشگاه علوم پزشکی تبریز انجام شد. افراد از گروه سنی 18 تا 24 سال انتخاب شدند. 25 نفر از شرکت‌کنندگان خانم و 25 نفر آقا بودند. آزمودنی‌ها به روش نمونه‌گیری آسان و غیرتصادفی انتخاب شدند. متغیرهای بررسی‌شده شامل میانگین سازه اول، دوم و سوم 6 واکه زبان فارسی در بافت کلمه است. همچنین ابزارهای استفاده‌شده در این پژوهش شامل پرسش‌نامه اطلاعات فردی (سن، جنس، دوزبانگی، وجود هرگونه بیماری مرتبط با سیستم تنفسی و گفتاری)، فرم رضایت‌نامه اخلاقی و یک دستگاه کامپیوتر مجهز به نرم‌افزار Pratt و یک دستگاه ضبط‌صوت سونی بود. پس از جمع‌آوری داده‌ها برای تحلیل آماری، به منظور مقایسه دو گروه در صورت برخوردار بودن از توزیع طبیعی از آزمون t مستقل و در صورت برخوردار نبودن از توزیع طبیعی از آزمون من ویتنی استفاده شد. تجزیه‌وتحلیل‌های آماری با استفاده از نرم‌افزار SPSS نسخه 18 انجام پذیرفت.  یافته‌ها: نتایج این پژوهش نشان داد بیشترین و کمترین مقدار F1 در آقایان و خانم‌ها مربوط به واکه‌های /æ/ و /i/ است. بیشترین و کمترین مقدار F2 در هر دو جنس مربوط به واکه‌های /i/ و/o/ است و بیشترین و کمترین مقدار F3 در آقایان مربوط به واکه‌های/i/ و /æ/ و در خانم‌ها مربوط به واکه‌های /i/ و /a/ در سطح کلمه است.  نتیجه‌گیری: با توجه به نتایج این مطالعه، واکه‌های /æ/ و /i/ در هر دو جنس به ترتیب بازترین (بیشترین F1) و بسته‌ترین واکه‌ها (کمترین F1) هستند. واکه‌های /i/ و/o/ در هر دو جنس به ترتیب پیشین‌ترین (بیشترین F2) و پسین‌ترین (کمترین F2) واکه‌ها هستند. گسترده‌ترین (بیشترین F3) واکه در آقایان و خانم‌ها مربوط به واکه /i/ و گردترین (کمترین F3) واکه در آقایان /æ/ و خانم‌ها /a/ است.
